# Lipopolysaccharide and lipotheicoic acid differentially modulate epididymal cytokine and chemokine profiles and sperm parameters in experimental acute epididymitis

**DOI:** 10.1038/s41598-017-17944-4

**Published:** 2018-01-08

**Authors:** Erick J. R. Silva, Camilla M. Ribeiro, André F. M. Mirim, Alan A. S. Silva, Renata M. Romano, Jorge Hallak, Maria Christina W. Avellar

**Affiliations:** 1 Section of Experimental Endocrinology, Department of Pharmacology, Universidade Federal de São Paulo–Escola Paulista de Medicina, São Paulo, SP 04044-020 Brazil; 2 Department of Pharmacology, Institute of Biosciences of Botucatu, Universidade Estadual Paulista “Júlio de Mesquita Filho”, Botucatu, SP 18618-869 Brazil; 3 Androscience, Science and Innovation Center in Andrology, São Paulo, SP 03178-200 Brazil; 4 Reproductive Toxicology Unity, Department of Pathology and Division of Urology, Hospital das Clínicas, University of São Paulo Medical School, São Paulo, SP 01246-903 Brazil; 5 Present Address: Department of Pharmacy, State University of Centro-Oeste, Guarapuava, PR 85040-080 Brazil

## Abstract

Bacterial infections are the most prevalent etiological factors of epididymitis, a commonly diagnosed inflammatory disease in the investigation of male infertility factors. The influence of early pathogenic mechanisms at play during bacterial epididymitis on reproductive outcomes is little understood. We report here that experimental epididymitis induced in rats by Gram-negative (LPS) and Gram-positive (LTA) bacterial products resulted in differential patterns of acute inflammation in the cauda epididymis. LPS elicited a strong inflammatory reaction, as reflected by upregulation of levels of mRNA for seven inflammatory mediators (*Il1b*, *Tnf*, *Il6*, *Ifng*, *Il10*, *Nos2* and *Nfkbia*), and tissue concentration of six cytokines/chemokines (IL1A, IL1B, IL6, IL10, CXCL2 and CCL2) within the first 24 h post-treatment. Conversely, LTA induced downregulation of one (*Nfkbia*) and upregulation of six (*Il1b*, *Il6*, *Nos2*, *Il4* *Il10* and *Ptgs1*) inflammatory gene transcripts, whereas increased the tissue concentration of three cytokines/chemokines (IL10, CXCL2 and CCL2). The stronger acute inflammatory response induced by LPS correlated with a reduction of epididymal sperm count and transit time that occurred at 1, 7, and 15 days post-treatment. Our study provides evidence that early epididymal inflammatory signaling events to bacterial activators of innate immunity may contribute to the detrimental effects of epididymitis upon male fertility.

## Introduction

The detection of pathogen-associated molecular patterns (PAMPs) by the innate immune system triggers host defence responses that are crucial to combat infection. The recognition of these PAMPs is made upon their binding to pattern recognition receptors (PRRs), which activate downstream signaling pathways initiating inflammation and other defence mechanisms, which in turn orchestrate antigen-specific adaptive immune responses^1,2^. Among the PRRs, the Toll-like receptor (TLR) family, which comprise 13 highly conserved transmembrane receptors that bind to PAMPs from a variety of pathogens, is the most studied in mammals^2^. In addition to their central role in the detection of pathogens by host cells, TLRs also bind to damage-associated molecular patterns (DAMPs) from dying or injured cells. Accumulating evidence indicates that these receptors facilitate the elimination of pathogens; additionally, they also participate in the development of chronic diseases such as cancer and metabolic syndrome, as well as other inflammatory conditions.

The interactions between the innate immune system and the male reproductive tract are crucial for the development and maintenance of male fertility^3^. The immune environment in the epididymis, a mucosal organ which plays crucial roles in sperm maturation, protection, and storage, must be tightly coordinated in order to rapidly mitigate pathogenic threats that could harm maturing spermatozoa. This milieu must also maintain a nurturing, yet tolerogenic environment to avoid the destruction of the auto-antigenic spermatozoon by the host immune system^3^. Although these immune mechanisms are crucial in keeping epididymal homeostasis, they remain largely unknown. As part of its localized innate immune system, the epididymis expresses several TLRs^3^, including TLR4 and TLR2/TLR6, which are activated by lipopolysaccharide (LPS) from Gram-negative bacteria and lipoteichoic acid (LTA) from Gram-positive bacteria, respectively. Under normal physiological conditions, these TLRs are found in epididymal epithelial and interstitial cells, and on luminal spermatozoa^4–6^. We demonstrated that following *in vivo* or *in vitro* exposure to *Escherichia coli*-derived LPS, the rat epididymis mounts an inflammatory response mediated by TLR4-dependent activation of the canonical nuclear factor kappa B (NFKB) transcription factor signaling pathway^5^. Likewise, primary *in vitro* cultures of rat epididymal epithelial cells have been shown to respond to the Gram-positive bacterium *Staphylococcus aureus* through TLR2 activation and subsequent induction of p38 MAPK and NFKB downstream signaling pathways^7^. Little is known, however, about the role of TLRs during the switch from homeostasis to an acute inflammatory condition upon epididymal bacterial infection.

Understanding how the epididymis senses and mounts defence responses against invading bacteria is of utmost clinical relevance because bacterial infections constitute the most prevalent etiology of epididymitis, an inflammatory condition commonly diagnosed in the investigation of male reproductive health and infertility factors^8^. Bacterial epididymitis may induce epididymal dysfunction, ultimately leading to temporary or persistent infertility^9,10^. It represents a serious threat to men’s health, especially during reproductive age, and can cause labor-hour losses, thus resulting in significant economic and public health issues^11,12^. Despite the deleterious effects of epididymitis on male fertility and well-being, the pathogen-specific reproductive outcomes of bacterial epididymitis are still scarcely understood^9,10,13^. In addition, most patients receive inadequate early diagnostic and therapeutic interventions, thus justifying the need for more basic and clinical research on this condition^14,15^.

*E. coli* (Gram-negative bacteria) and *Enterococcus spp* (Gram-positive bacteria) are among the most prevalent causative pathogens of epididymitis^8,12^. These pathogens mostly reach and colonize the epididymis via their retrograde ascent through the urethra^8^. The retrograde injection of bacteria into the vas deferens lumen from rodents, an experimental approach that mimics clinical infectious epididymitis, renders a dysfunctional epididymis^8^. Intravasal *E. coli*-induced epididymitis in rats and mice revealed striking morphological changes that include epithelial and smooth muscle damage, ductal obstruction, and fibrotic remodeling, mainly in the distal region of the epididymis (cauda epididymis), which also displayed significant changes in gene expression and cell death pathways^10,13,16–23^. Most of these parameters, however, were evaluated several days after inducing epididymitis^8^. Thus, the earliest *in vivo* events of epididymal TLR-mediated signaling upon recognition of luminal bacterial PAMPs are yet to be determined. Unraveling these events in a temporal, yet mechanistic fashion will better elucidate the pathogenic process of bacterial epididymitis and its consequent impact on epididymal function. These studies could, in turn, facilitate the identification of targets for adjuvant therapy of this disease as possible tools to minimize its detrimental effects on fertility.

In the present study, we tested the hypothesis that the differential activation of TLR4 and TLR2/TLR6 by LPS and LTA, respectively, in the epididymis induces a differential pattern of acute inflammation, which may influence the natural history of epididymitis as well as the severity of its reproductive outcomes. To that end, we evaluated the early effects of epididymitis induced by the retrograde intravasal injection of either ultrapurified LPS from *E. coli* or LTA from *E. faecalis* on both the expression of inflammatory genes and cytokine/chemokine tissue concentration in the cauda epididymis. We have also determined the effects of LPS- and LTA-induced epididymitis on plasma steroid hormone levels, as well as testicular and epididymal sperm parameters.

## Results

### Intravasal injection of LPS or LTA differentially modulated the mRNA levels of pro- and anti-inflammatory genes in the rat cauda epididymis

We took advantage of the rat model of epididymitis induced by the retrograde injection of PAMPs from Gram-negative and Gram-positive bacteria into the lumen of the vas deferens to study their differential effects on the epididymis *in vivo*. This experimental model has been widely used to study the pathogenic mechanisms at play during bacterial epididymitis^10,16,18,21,24,25^, because it simulates the clinical condition in which bacteria reach and colonize the epididymis via retrograde ascension. Thus, we explored this experimental model to directly challenge the epididymis with bacterial-derived agonists of TLR4 (LPS) and TLR2/TLR6 (LTA) *in vivo* via luminal environment. The rodent epididymis (both rat and mouse) is highly segmented by connective tissue septa^26^ and it has been demonstrated that these interstitial structures provide physical barriers to ascending pathogens^19^. By performing intravasal injections with Blue Evans dye, we corroborated these findings and observed that the ascent of the dye was limited to the cauda epididymis 30 min post-treatment (see Supplementary Results; Supplementary Fig. S1).

**Figure 1:**
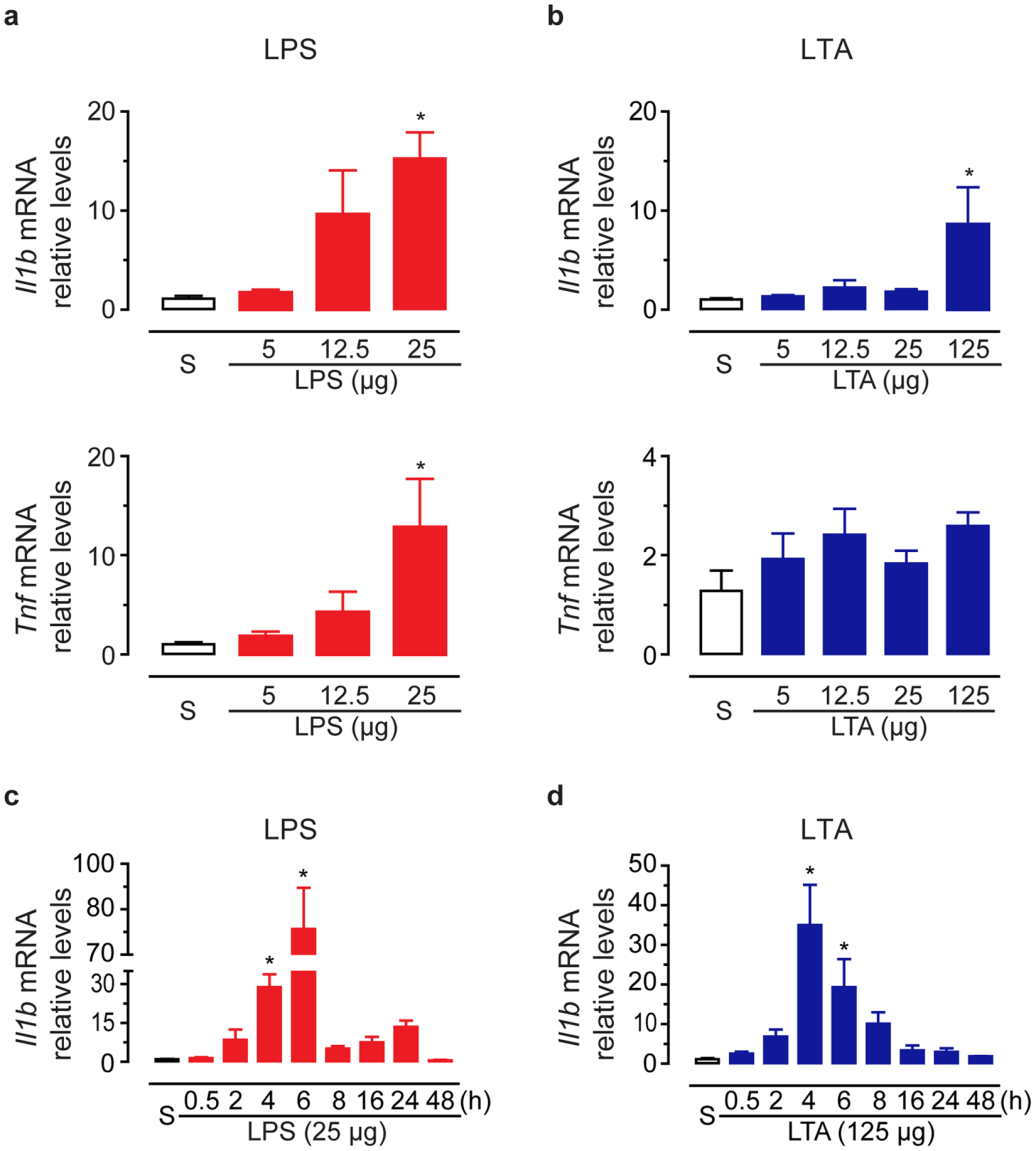
Effects of intravasal LPS or LTA treatment on the mRNA levels of the pro-inflammatory gene markers interleukin-1beta (*Il1b*) and tumoral necrosis factor-alpha (*Tnf*). (**a**,**b**) Relative quantification of *Il1b* and *Tnf* transcripts by RT-qPCR in the cauda epididymis from saline-control (S), LPS- or LTA-treated rats. Tissues were collected 6 h after treatment from three (5 μg and 12.5 μg LTA) or four (saline-control; 5, 12.5 and 25 μg LPS; 25 and 125 μg LTA) rats per group. (**c**,**d**) Relative quantification of *Il1b* transcript levels by RT-qPCR in the cauda epididymis from saline-control (S), LPS(25 μg)- or LTA(125 μg)-treated rats at the indicated time points. Tissues were collected from three (8-h LTA), four (saline-control; all LPS time-points; and 0.5-h, 2-h, 4-h, and 16-h LTA) or five (24-h and 48-h LTA) rats per group. Data points in (**a–d**) were normalized to their internal control (*Ppia*) and expressed as relative values of their respective saline-control groups. Asterisks indicate statistically different from saline-control group (p < 0.05, ANOVA followed by Dunnet’s test). Results are expressed as mean ± SEM of experiments performed in duplicate.

In order to examine the cauda epididymis responsiveness to PAMPs from Gram-negative and Gram-positive bacteria, we evaluated the acute inflammatory response 6 h after intravasal injection of different doses of both ultrapurified LPS from *E. coli* (5, 12.5 or 25 μg; TLR4 agonist) and LTA from *E. faecalis* (5, 12.5, 25 or 125 μg; TLR2/TLR6 agonist). We used the inflammatory markers *Il1b* and *Tnf mRNA level* quantification *by RT-qPCR* as a readout. We observed that neither 5 nor 12.5 μg LPS treatment changed mRNA basal levels of *Il1b* and *Tnf* (Fig. 1a). In contrast, 25 μg LPS increased both *Il1b* and *Tnf* transcript levels (fold-changes of 14.3 ± 2.6, and 12.8 ± 4.9, respectively) in comparison to saline-control (Fig. 1a). Intravasal injection of LTA upregulated *Il1b mRNA* levels in the cauda epididymis only at the highest dose tested (125 μg; fold-change of 8.6 ± 3.8) (Fig. 1b). Interestingly, *Tnf* transcript levels were similar among saline-control and LTA-treated groups (Fig. 1b).

Considering that 25 μg LPS and 125 μg LTA induced comparable changes on *Il1b* mRNA levels, we next evaluated the effects of these PAMPS at these doses on the time-course expression changes of *Il1b* transcripts in the cauda epididymis (Fig. 1c–d). We observed an upregulation of *Il1b* transcript levels as early as 4 h post LPS challenge in comparison to saline-control group, reaching a maximum peak at 6 h (fold-changes of 28.9 ± 5.0, and 78.5 ± 13.6, respectively) (Fig. 1c). We detected a second, but not significant peak of *Il1b* transcript levels (fold-change of 13.6 ± 2.4) 24 h after LPS challenge (Fig. 1c). In response to LTA, *Il1b* mRNA levels were also upregulated at 4 h and 6 h, but reached a maximum peak at 4 h post-treatment (fold-changes of 35.0 ± 10.1, and 19.3 ± 7.1, respectively) (Fig. 1d). The LTA-induced changes in *Il1b* mRNA levels exhibited a bell-shaped time-course pattern in the cauda epididymis, with no later second peak (Fig. 1d). Consistent with these findings, morphological analysis revealed similar displays of acute inflammation in the cauda epididymis following LPS or LTA intravasal treatments (see Supplementary results, Supplementary Figs S2, S3). Altogether, our findings demonstrated that the epididymis rapidly mounted an inflammatory response to either LPS or LTA *in vivo* intraluminal exposure. The differences in the LPS- and LTA-induced *Il1b* and *Tnf* expression profile, however, indicated differential epididymal sensitivity according to the inflammatory triggering stimulus.

**Figure 2:**
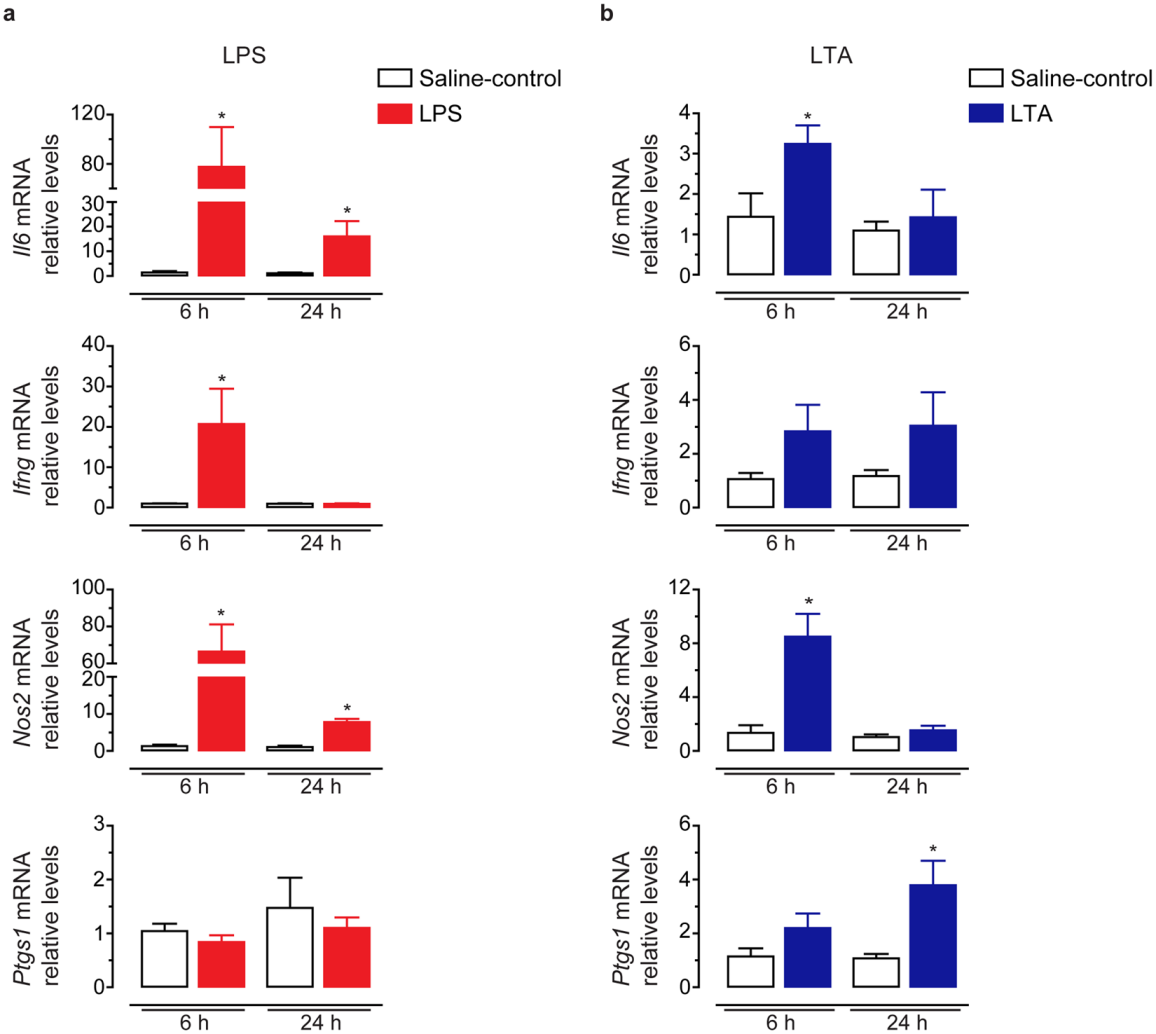
Effect of intravasal LPS (25 μg) or LTA (125 μg) treatment on the mRNA levels of pro-inflammatory genes in the cauda epididymis. (**a**,**b**) Relative quantification of *Il6*, *Ifng*, *Nos2* and *Ptgs1* transcripts by RT-qPCR in the cauda epididymis from saline-control, LPS- or LTA-treated rats euthanized 6 h or 24 h after treatment. Samples were normalized to their internal control (*Ppia*) and expressed as relative values of their respective saline-control groups. Asterisks indicate statistically different from saline-control group (p < 0.05, Student’s t test). Results are expressed as mean ± SEM of experiments performed in duplicate with samples from five (24-h saline-control) or six (6-h saline-control, LPS- and LTA-treated groups) rats per group in (**a**) and five (6-h saline-control and 24-h LTA) or six (24-h saline-control and 6-h LTA) rats per group in (**b**).

To address this hypothesis, we performed qPCR experiments to examine the relative transcript levels of pro-inflammatory (*Il6*, *Ifng*, *Ccl5*, *Nos2*, *Ptgs1* and *Ptgs2*) and anti-inflammatory (*Nfkbia*, *Il4* and *Il10*) mediators in the cauda epididymis 6 h and 24 h after the intravasal injection of LPS and LTA. Both LPS and LTA increased the mRNA levels of the pro-inflammatory cytokine *Il6* and the inflammatory marker *Nos2* at 6 h in comparison to saline-control groups (Fig. 2a,b). Remarkably, these effects on *Il6* and *Nos2* mRNA levels were more robust in tissues from LPS- than LTA-treated rats (Fig. 2a,b). *Il6* and *Nos2* transcript levels remained upregulated at 24 h only in the LPS-treated group (Fig. 2a). Furthermore, we observed that intravasal challenge with LPS, but not LTA, upregulated the mRNA levels of the Th1-type cytokine *Ifng* at 6 h (Fig. 2a,b). Conversely, we detected that only LTA treatment increased the mRNA levels of the cyclooxygenase enzyme *Ptgs1* (also known as *Cox1*) at 24 h (Fig. 2a,b). Neither LPS nor LTA treatment changed the mRNA levels of the chemokine *Ccl5* and the cyclooxygenase enzyme *Ptgs2* (see Supplementary Fig. S4).

**Figure 3:**
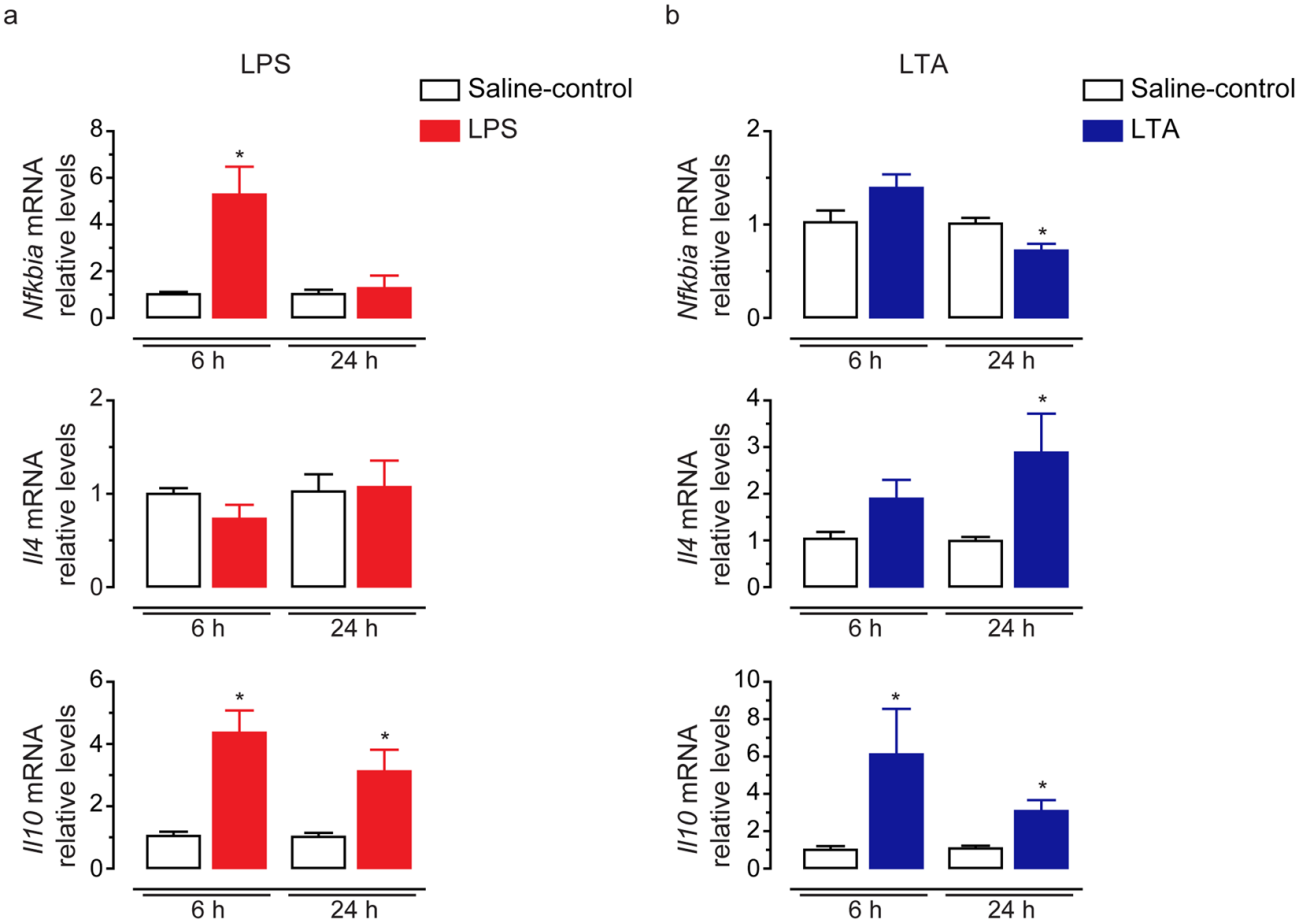
Effects of intravasal LPS (25 μg) or LTA (125 μg) treatment on the mRNA levels of anti-inflammatory genes in the cauda epididymis. (**a**,**b**) Relative quantification of *Il10*, *Il4* and *Nfkbia* transcripts by qPCR in the cauda epididymis from saline-control, LPS- or LTA-treated rats euthanized 6 h or 24 h after treatment. Samples were normalized to their internal control (*Ppia*) and expressed as relative values of their respective saline-control groups. Asterisks indicate statistically different from saline-control group (p < 0.05, Student’s t test). Results are expressed as mean ± SEM of experiments performed in duplicate with samples from six rats per group in (**a**) and five (24-h saline-control) or six (6-h and 24-h saline-control, 6-h and 24-h LTA) rats per group in (**b**).

The mRNA levels of anti-inflammatory mediators also exhibited a differential expression pattern depending on the type of inflammatory stimulus. We observed that intravasal LPS treatment increased the mRNA levels of the anti-inflammatory marker *Nfkbia* at 6 h (Fig. 3a), whereas LTA treatment downregulated this transcript at 24 h in comparison to their respective saline-control groups (Fig. 3b). We further detected that intravasal LTA, but not LPS treatment, upregulated the mRNA levels of the anti-inflammatory Th2-type cytokine *Il4* at 24 h (Fig. 3a,b). Both, LPS and LTA, upregulated the mRNA levels of the anti-inflammatory cytokine *Il10* at 6 h and 24 h (Fig. 3a,b).

### Intravasal LPS or LTA injection differentially modulated tissue concentration of cytokines and chemokines in the cauda epididymis

**Figure 4:**
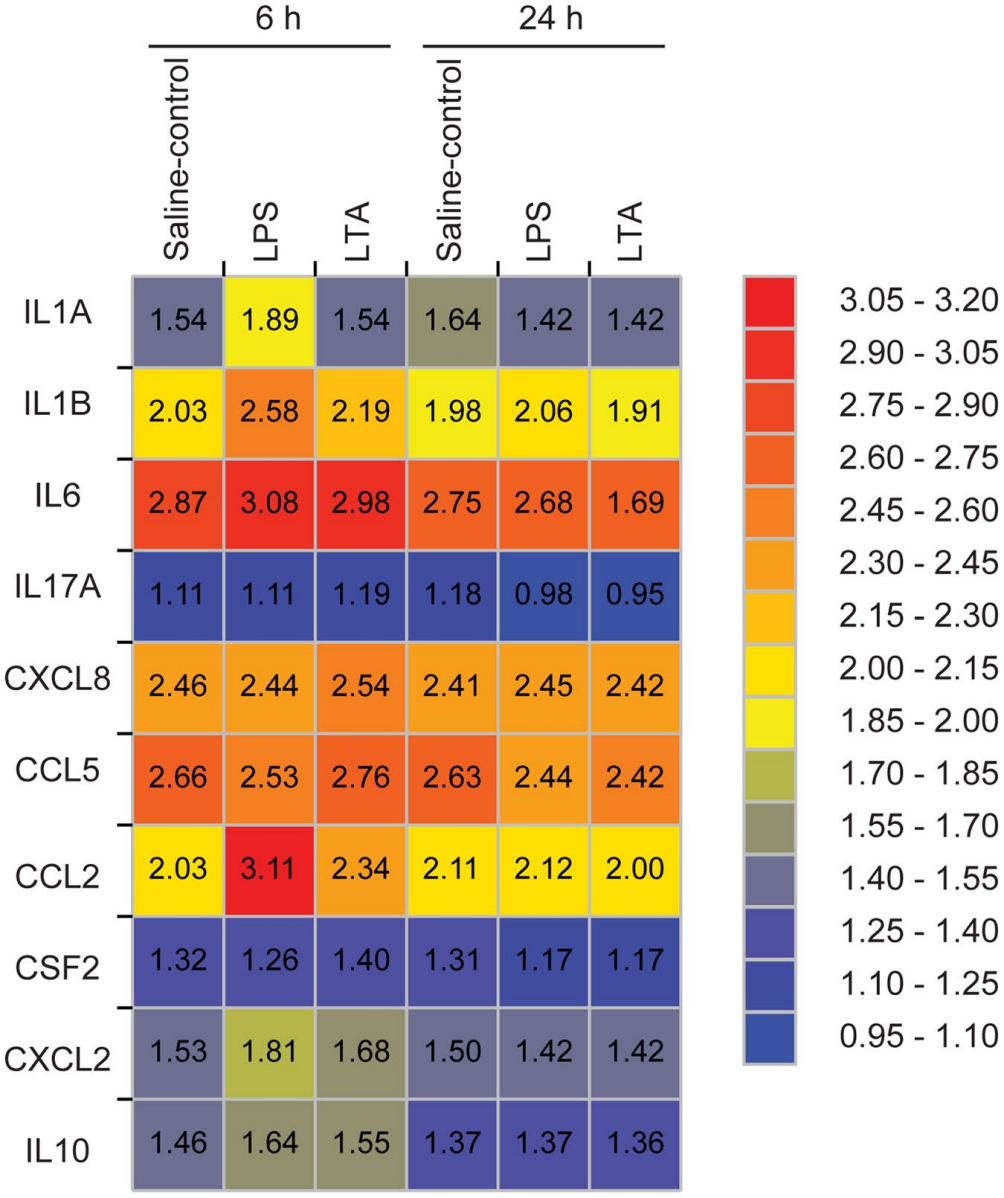
Heat map showing the effects of intravasal LPS (25 μg) or LTA (125 μg) treatment on the cytokine and chemokine expression profiles in the cauda epididymis. Tissues were collected from saline-control, LPS- or LTA-treated rats euthanized 6 h or 24 h post-treatment. Values represent Log_10_ from the average values shown in Supplementary Table S1 (average cytokine or chemokine concentration, pg of target protein/mg of tissue total protein). Blue indicates the lowest levels, yellow indicates median levels, and red indicates the highest levels.

To further understand the inflammatory environment in the epididymis upon LPS and LTA luminal exposure, we evaluated their effects on the concentration of a set of five cytokines (IL1A, IL1B, IL6, IL17A, and IL10) and five chemokines (CXCL8, CCL2, CXCL2, CSF2, and CCL5) in protein extracts from the cauda epididymis (Fig. 4 and Supplementary Table S1). Notably, we detected basal levels of all these mediators in the intact rat cauda epididymis, as follows (mean ± SEM pg/mg of protein, n = 6 rats): IL1A (22.3 ± 2.1), IL1B (79.2 ± 5.5), IL6 (500.3 ± 44.8), IL17A (10.9 ± 1.1), IL10 (21.5 ± 2.3), CXCL8 (284.9 ± 17.2), CCL2 (60.2 ± 4.8), CXCL2 (28.0 ± 2.7), CSF2 (17.5 ± 1.6), and CCL5 (331.4 ± 26.7). We plotted the time-dependent changes in the cytokine/chemokine profile in the cauda epididymis from saline-control versus LPS or LTA-treated rats in a heat map (Fig. 4). Among the targets analyzed, we showed that intravasal LPS treatment increased tissue concentrations of four cytokines and two chemokines at 6 h in comparison to saline-control group. LTA, on the other hand, induced upregulation of only one cytokine and two chemokines at the same time-point (Fig. 4 and Supplementary Table S1).

LPS treatment increased the tissue levels of the acute phase pro-inflammatory cytokines IL1A (2.2 fold-change), IL1B (3.0 fold-change), IL6 (1.9 fold-change), as well as the chemokines CCL2 (12.5 fold-change) and CXCL2 (1.9 fold-change) (Fig. 4 and Supplementary Table S1). LTA treatment, on the other hand, upregulated CCL2 (2.2 fold-change) and CXCL2 (1.8 fold-change) tissue concentrations, but did not affect IL1A, IL1B and IL6 production (Fig. 4 and Supplementary Table S1). Furthermore, LPS and LTA treatments increased tissue concentration of the anti-inflammatory cytokine IL10 (1.5 fold-change for LPS and LTA) (Fig. 4 and Supplementary Table S1). Neither LPS nor LTA treatment changed the basal tissue levels of IL17A, CXCL8, CCL5, and CSF2 (Fig. 4 and Supplementary Table S1). At 24 h post-treatment, we detected that all tested cytokines and chemokines returned to similar concentrations observed in the cauda epididymis from saline-control rats (Fig. 4 and Supplementary Table S1). Altogether, our data suggest that the cauda epididymis differently responds to intraluminal LPS and LTA by upregulating unique respective sets of inflammatory genes.

**Figure 5:**
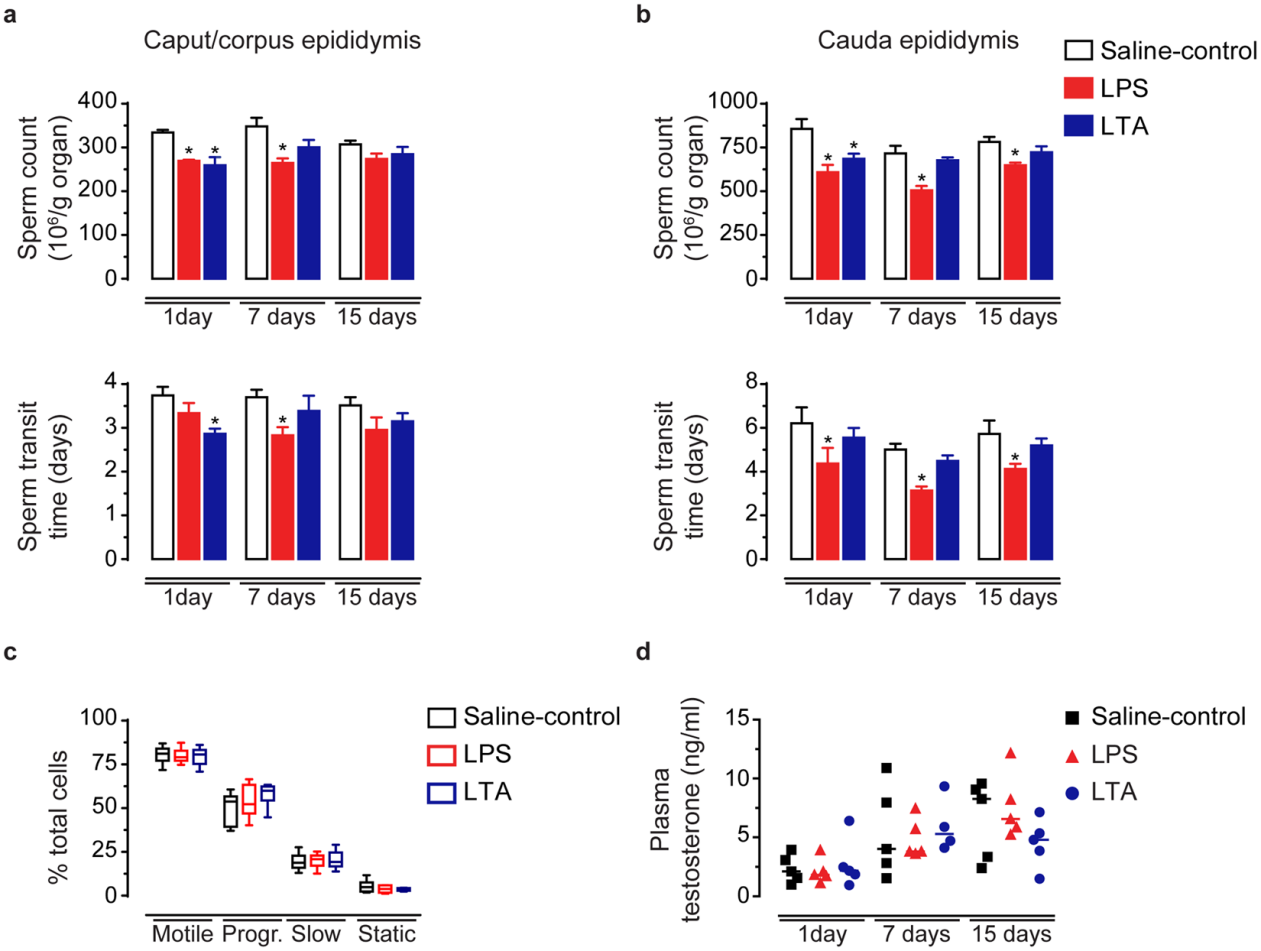
Effects of intravasal LPS (25 μg) or LTA (125 μg) treatment on epididymal sperm parameters and plasma testosterone levels. (**a**,**b**) Sperm count and transit time in the caput/corpus and cauda epididymis from saline-control, LPS- or LTA-treated rats euthanized 1, 7 or 15 days post-treatment. Asterisks indicate statistically different from their respective saline-control group (ANOVA followed by Bonferroni test, p < 0.05). Results are expressed as mean ± SEM of experiments performed with samples from six (1-day LPS and 1-day LTA), seven (1-day saline-control), and eight (7− and 15-day saline-control, 7− and 15-day LPS, and 7− and 15-day LTA) rats per group. (**c**) Motility of cauda epididymis sperm from saline-control, LPS- or LTA-treated rats euthanized 7 days post-treatment analyzed by CASA. Box plots (median, interquartile range and whiskers min-max) represent the percentage of motile, progressive and static spermatozoa. Experiments were performed with samples from six rats per group. (**d**) Testosterone concentration in plasma samples from the same experimental groups. Each symbol represents one individual plasma sample from saline-control (square), LPS-(triangle) and LTA-(circle) treated rats, as indicated. The  horizontal line is the median value to each group.

### Intravasal LPS or LTA injection did not affect plasma testosterone levels, testicular sperm count and sperm motility, but impaired epididymal sperm number and transit time in an region-specific fashion

From our gene expression data we raised the question whether differences in the epididymal inflammatory reaction to LPS and LTA could result in different reproductive outcomes. Since the crucial role of the epididymis to reproduction is to support sperm maturation, protection and storage prior ejaculation, we addressed this topic by evaluating qualitative and quantitative sperm parameters after inducing epididymitis with LPS and LTA. We detected that intravasal LPS and LTA treatment did not affect plasma testosterone levels (Fig. 5d) or testicular sperm count and daily sperm production (see Supplementary Fig. S5). When compared to saline-control groups, however, we observed lower sperm counts in the caput/corpus epididymis at 1 and 7 days after LPS treatment, and in the cauda region at 1, 7 and 15 days (Fig. 5a,b). In parallel, we observed an acceleration of the sperm transit time in the caput/corpus epididymis at 7 days after LPS treatment. In the cauda region, however, intravasal LPS increased sperm transit time at all treatment time-points analyzed, which was indicative of a faster sperm transport along this epididymal region for up to two weeks after treatment (Fig. 5a,b). Conversely, LTA-induced epididymitis reduced sperm count in both caput/corpus and cauda epididymis, but only at 1 day post-treatment (Fig. 5a). At this time-point, we found an acceleration of sperm transit time in the caput/corpus epididymis (Fig. 5a). Interestingly, neither LPS nor LTA affected motility (Fig. 5c and Supplementary Table S2) and morphology (Supplementary Fig. S6) of spermatozoa collected from the cauda epididymis 7 days after inducing epididymitis.

## Discussion

Epididymitis represents the most frequent cause of scrotal pain and inflammation^8,27^, imposing serious threats to male fertility and well-being. Bacterial infections ascending through the urethra are responsible for the majority of epididymitis cases^8,12^. Here we used a rat experimental model of epididymitis to study the local acute inflammatory responses of the epididymis to either ultrapurified LPS from *E. coli* or LTA from *E. faecalis*, two frequent causative pathogens of epididymitis^8,12^. The use of these purified PAMPs as pharmacological inducers of inflammation allowed us to more precisely define the events triggering the TLR4- and TLR2/TLR6-dependent inflammatory responses induced by LPS and LTA, respectively. We found that LPS and LTA luminal challenge rapidly induced an inflammatory reaction in the cauda epididymis by upregulating distinct respective sets of inflammatory mediators. The more robust inflammatory response of the epididymis to LPS when compared to LTA correlated well with the more extensive LPS effects on epididymal sperm parameters. Taken together, our results demonstrate that the epididymis can differentially recognize PAMPs from Gram-negative and Gram-positive bacteria, shedding new light into the understanding of the early inflammatory events during bacterial epididymitis. Our study also highlights the importance of early innate immune mediators to the natural history of bacterial epididymitis and its impact on fertility.

Considering that the epididymis is an aseptic site, it has been suggested that its epithelial cells are in an active surveillance state to promptly detect and respond to potential luminal pathogens^8,28^. Our study supports this hypothesis since the expression of TLR4 and TLR2/TLR6 by epididymal cells may trigger inflammatory responses to luminal LPS and LTA, respectively. Despite triggering morphologically similar displays of acute inflammation, we found that LPS and LTA induced distinct expression profiles (at mRNA and protein levels) of inflammatory mediators, which suggested different immune mechanisms may be at play during epididymitis induced by Gram-negative and Gram-positive bacteria. Our results showed that LPS stimulated an inflammatory response characterized by potent induction of mRNA expression for pro-inflammatory cytokines, such as *Il1b*, *Tnf*, *Il6*, and *Ifng*. Consistent with our mRNA data, LPS increased the tissue concentrations of IL1B, IL6, and IL10, as well as IL1A, CCL2 and CXCL2, which may play important roles in immune cell recruitment to the epididymis upon infection by Gram-negative bacteria. LTA challenge, on the other hand, presented less extensive effects on the mRNA expression of inflammatory mediators and the secretion of cytokines and chemokines in comparison to LPS. The lack of IL1B release after LTA treatment in the cauda epididymis suggested that, unlike LPS, LTA was unable to induce maturation of IL1B from its inactive precursor, which thereby could hinder the secretion of its downstream cytokine IL6^29^. Cooperation between *Il10* and *Il4*, both genes upregulated by LTA in the cauda epididymis, could have contributed to the less robust effects of this PAMP on inducing pro-inflammatory gene expression when compared to LPS. In fact, it has been demonstrated that IL10 and IL4 act synergistically to induce immunosuppression in mice treated with helminth-derived molecules^30^.

The differences in inflammatory gene expression induced by LPS and LTA in the cauda epididymis could be due to differences in TLR4 and TLR2/TLR6 signaling pathways. It is known that activation of TLR4 triggers both the myeloid-differentiation primary response 88 (MYD88)-dependent pathway and the TLR adaptor molecule 1 (TICAM1)-dependent pathway, leading to the activation of the NFKB, IRF, and MAPK pathways^31^. Activation of TLR2, in turn, results exclusively in the activation of the MYD88-dependent pathway, which may differently impact the functional states of signaling molecules in comparison to the TLR4 signaling cascade^31–33^. Differential effects of LPS and LTA on the activation of MAPK (p38 and ERK), AP1 and NFKB pathways have been demonstrated in monocytes isolated from adult and neonatal humans^34^. Consistently, we observed a differential effect of LPS and LTA on the expression of classical NFKB-regulated genes, such as *Nfkbia*, *Il1b*, and *Tnf*. We have previously demonstrated that intravenous LPS treatment led to NFKB activation mainly as RELA/RELA homodimers in the rat epididymis^5^, rather than the most commonly activated RELA/NFKB1 heterodimers^35^. Data from our study encourage new experiments to evaluate whether activation of TLR4 and TLR2/TLR6 signaling pathways by intraluminal LPS and LTA, respectively, could induce differential activation of NFKB hetero- and homodimers in the epididymis, as a molecular mechanism by which these PAMPs modulate the epididymal immune environment.

In several organs, cyclooxygenase (PTGS) enzymes show an expression pattern characterized by the constitutive expression of PTGS1 and the inducible expression of PTGS2 by inflammatory stimuli. In the rodent epididymis, however, both PTGS1 and PTGS2 are expressed under normal conditions^36^. Our results confirmed this observation and further showed that neither LPS nor LTA were capable of inducing *Ptgs2* expression in the cauda epididymis, most likely due to its high steady-state levels under normal conditions^36^. The upregulation of *Ptgs1* transcript levels after LTA treatment suggests a potential role for PTGS1 in the epididymal immune response to Gram-positive bacteria. In fact, recent findings indicated that increased expression of PTGS1 in the epididymis of the indoleamine 2,3-dioxygenase 1 knock-out (*Ido* ^*−/−*^) mouse facilitates inflammation via engagement of the Th17 immune response^37^. It is worth noting that PTGS1 is primarily expressed in the basal cells of the epididymal epithelium, where it plays important roles as a mediating molecule in the cross-talk among epididymal epithelial cell types^38^. Both TLR2 and TLR6 are expressed in basal cells from the rat epididymis^6^. Thus, it is likely that LTA present in the lumen of epididymis may regulate *Ptgs1* expression by directly activating TLR2/TLR6 on basal cells. This hypothesis opens a new avenue of investigation on the roles of cyclooxygenases in the inflamed epididymis, which may provide new tools for the rational use of non-steroidal anti-inflammatory drugs as adjuvant treatment of epididymitis.

Bacterial epididymitis may cause epididymal duct obstruction, epithelial and smooth muscle tissue damage and fibrotic remodeling, thus leading to infertility^13,19^. It has been suggested that the severity of epididymal damage could be related to the pattern of inflammatory and immune responses elicited upon bacterial infection^10,13^. Accordingly, our results indicated that differences in the early inflammatory responses to specific PAMPs are relevant to the prognosis of epididymitis-induced fertility outcomes, as LPS caused more drastic and longer effects on sperm number and transit time along the epididymis, while LTA effects were shorter and milder. The acute inflammation caused by LPS impaired sperm parameters along the entire epididymis as demonstrated by decreased sperm count and transit time in both proximal and distal epididymal regions. Changes in the contractility of the epididymal duct, which is under the control of neuronal (e.g. autonomic innervation), hormonal (e.g. androgens) and paracrine (e.g. prostaglandins) factors, could play a role in the acceleration of sperm transit time in the epididymis of LPS-treated rats, an event that is correlated with poor sperm maturation and reduced fertilizing capacity^39^. Lang *et al*.^10^ showed that sperm count in the semen of epididymitis patients infected with non-uropathogenic (NPEC) and uropathogenic (UPEC) *E. coli* strains was reduced 14 days after antibiotic treatment. Sperm count remained lower after 3 months only in UPEC*-*infected patients, an effect attributed to the UPEC virulence factor haemolysin^10^. Our results suggest that LPS contributes to sperm count decrease during the early stages of *E. coli* infection, which in turn induce short-term oligospermia in acute epididymitis subjects, while other *E. coli* virulence factors are responsible for the long-term detrimental effects on sperm parameters.

Testicular sperm parameters and plasma testosterone levels did not change in LPS-treated rats, thus indicating that LPS effects on epididymal sperm parameters were due to disturbances in the epididymis environment itself rather than secondary to spermatogenesis and/or testicular steroidogenesis impairment. This hypothesis is supported by our data, which indicated a reduction of sperm number in the cauda epididymis by 24 h post LPS challenge, and by the fact that this endotoxin-induced apoptosis in human and mouse sperm *in vitro* ^40,41^, an effect not observed in spermatozoa from the TLR4 knock-out mouse^41^. In addition, incubation of human spermatozoa with pro-inflammatory cytokines, such as TNF and IL6, activated apoptotic mechanisms in a similar time frame^42–44^. Consistently, we observed increased expression of these cytokines in the epididymis from LPS-, but not LTA-treated animals. Whether apoptosis of spermatozoa during acute epididymitis could contribute to reduction of epididymal sperm number *in vivo*, however, remains to be determined. Thus, the harmful effects of LPS-induced epididymitis on epididymal sperm parameters could be due to both direct cytotoxic effects of LPS on spermatozoa through TLR4-dependent pathways and/or indirectly via cytokine secretion and ROS production by the immune and non-immune cells of the epididymis.

In conclusion, we showed that the epididymis is able to recruit different elements of the innate immune system in a PAMP-specific manner *in vivo*. The understanding of the onset of the epididymal inflammatory reaction upon an infection by Gram-negative or Gram-positive bacteria can be determinant for correct early diagnosis and adjuvant therapy, which could help in preventing chronic lesions that lead to irreversible loss of fertility and chronic scrotal pain. The intensity of the early inflammatory events may be considered a contributing factor to the severity of bacterial epididymitis and its subsequent detrimental effects on fertility.

## Methods

### Chemicals

Reagents and chemicals were molecular biology grade from Sigma (St. Louis, MO, USA) or Life Technologies (Carlsbad, CA, USA). Ultrapure LPS from *E. coli* 055:B5 (S-form; purity ≥ 99.9%, 1 ng = 10 endotoxin units) was from Innaxon (Oakield Close, UK). LTA from *E. faecalis* was from Sigma (St. Louis, MO, USA).

### Animals

Adult male Wistar rats (90 days old) were housed in the Animal Facility at Instituto Nacional de Farmacologia (INFAR), Universidade Federal de São Paulo – Escola Paulista de Medicina (Unifesp-EPM) and maintained under controlled light (12 h light:dark cycle) and temperature (22–24 °C), with food and water *ad libitum*. Animals were manipulated daily during the week before surgery to reduce stress levels. Animal procedures were conducted according to guidelines for animal care and use of laboratory animal, approved by Unifesp-EPM Research Ethics Committee (CEP #0310/12).

### Animal model of epididymitis

Rats were anesthetized with ketamine/xylazin (100 and 10 mg/kg, i.p.). A single scrotal incision was made to expose the epididymal portion of the vas deferens. The vas deferens was clamped with a hemostatic clamp and 25 μl of sterile saline containing different doses of LPS (5, 12.5, and 25 μg) or LTA (5, 12.5, 25, and 125 μg) were injected into the vas deferens using a 30-G needle and a 0.1-ml Hamilton syringe. LPS doses were equivalent to 50,000 (5 μg), 125,000 (12.5 μg) and 250,000 (25 μg) endotoxin units (EU). The injection was made bilaterally in a retrograde direction as near as possible to the cauda epididymis. Sham-operated rats underwent a similar procedure, but were treated with sterile saline only (saline-control). Another control group consisted of rats that did not undergo any procedure. Rats were euthanized at different post-treatment time points (0.5 to 48 h for mRNA, protein and histological studies; 1 to 15 days for sperm count evaluation and hormone assays). Testes and epididymides were dissected, weighed and processed for histological and molecular studies. Blood samples were collected into EDTA-treated tubes immediately after euthanasia. After centrifugation (1,000 × g, 15 min), plasma samples were aliquoted and stored at −20 °C until use for testosterone measurement.

### Reverse transcriptase-quantitative polymerase chain reaction (RT-qPCR)

Frozen cauda epididymides from saline-control, LPS- and LTA-treated rats were pulverized in liquid nitrogen and used for total RNA extraction using Trizol® (Invitrogen). Total RNA (2.5 μg) was reverse transcribed using Thermoscript^TM^ RT-PCR system (Invitrogen). cDNA samples were assayed in qPCR performed with SYBR Green Master Kit (Kapa Biosystems, Cape Town, South Africa) in a 7500 Real-Time PCR System (Applied Biosystems, Foster City, CA, USA). Reactions were run in duplicates and samples with no cDNA template were used as negative control. Amplifications were carried out using specific primers for each target (see Supplementary Table S2) under the following cycle conditions: 50 °C (2 min), 95 °C (3 min) and 40 cycles of 95 °C (15 s) and 60 °C (1 min). At the end of the reaction, a melting curve was generated and analyzed to confirm the specificity of the amplification. Routinely, end-point PCR products were analyzed by 1.5% agarose gel electrophoresis. The cycle threshold (Ct) for each replicate was determined using 7500 Applied Biosystems software. The amplification efficiency was determined for each set of primers from Ct values generated by serially diluted cDNA samples (see Supplementary Table S3). Relative expression of each target gene was normalized with the reference gene cyclophilin A (*Ppia*), used as endogenous control and calculated using the formula described by Pfaffl^45^, which includes the experimentally determined amplification efficiency. The Ct values for the reference gene were stable among samples (*Ppia* Ct variation values for data shown in Figs 1, 3 and 4: standard deviation ≤0.45, coefficient of variation ≤2.48%, n = 16–40). Data were expressed as relative values of the saline-control groups, which were obtained in parallel to each LPS- and LTA-treated groups.

### Cytokine and chemokine multiplex bead assay

Total protein extracts of cauda epididymis from intact, saline-control, LPS- and LTA-treated rats were prepared. Frozen cauda epididymides (∼100 mg) were pulverized in liquid nitrogen and dounce-homogenized in 3 volumes of lysis buffer (9.1 mM NaH_2_PO4, 1.7 mM Na_2_HPO4, 150 mM NaCl, 0.5% Tween-20, containing 80 μM leupeptin, 1.5 μM aprotinin, 29 μM pepstatin A, 1 mM phenylmethylsulfonil fluoride, and 0.5 mM pefabloc). After 30-min incubation on ice, samples were centrifuged twice (10,000 × g, 4 °C, 10 min). Supernatants were aliquoted and stored at −75 °C until use. Total protein concentration was determined with BioRad protein assay reagent (BioRad Laboratories, Richmond, CA, USA), using bovine serum albumin as standard.

Tissue concentrations of IL1A, IL1B, IL6, IL10, IL17A, CXCL8 (IL18), CSF2 (GMCSF), CCL2 (MCP1), CXCL2 (MIP2) and CCL5 (RANTES) were simultaneously determined by using a magnetic bead assay (Milliplex MAP Rat Cytokine/Chemokyne Panel, Millipore, Billerica, MA, USA). All procedures were performed according to manufacturer’s instructions, at room temperature and protected from light. Samples were analyzed in a Luminex MAGPIX system (Millipore). Cytokines and chemokines concentrations in each epididymal sample were calculated using Analyst software (Millipore) with a five-parameter logistic curve-fitting method. Data were normalized to the amount of total protein in each epididymal sample and expressed as μg of target protein/mg of tissue total protein. Normalized cytokine or chemokine concentrations were transformed to their Log_10_ values and the averaged data used to generate a heat map with JColorGrid software^46^.

### Histopathological evaluation

Whole epididymides from saline-control, LPS- and LTA-treated rats were immersed in Bouin’s fixative (75 ml saturated picric acid, 5 ml glacial acetic acid, 25 ml 37% formaldehyde) overnight and processed for paraffin embedding. Two non-consecutive longitudinal tissue sections (5 µm) were stained with hematoxylin/eosin, and observed for general histopathological examination under light microscope. Images were acquired using a digital camera and Image-Pro Express Software (Media Cybernetics, Silver Spring, MD, USA). Samples were evaluated by two investigators blinded to the study.

### Testicular and epididymal sperm parameters

Sperm count was performed in the testis and epididymis (caput/corpus and cauda regions) from saline-control, LPS- and LTA-treated rats. Homogenization-resistant testicular spermatid quantification (stage 17–19 of spermiogenesis) was performed as described by Robb *et al*.^47^. Briefly, decapsulated testes were homogenized in ST solution (0.9% NaCl, 0.05% Triton X-100) and sonicated. Samples were diluted in ST solution and transferred to a Neubauer hemocytometer for sperm count. Daily sperm production (DSP) was calculated by dividing the number of homogenization-resistant spermatids by 6.1 (i.e. the number of days that these cells are present in the seminiferous epithelium in the rat). Caput/corpus and cauda epididymides were processed for sperm count as described above. Sperm count and DSP were expressed as 10^6^ cells/g of tissue. Epididymal sperm transit time was expressed in days; it was calculated by dividing sperm count within each epididymal region by DSP.

Analysis of motility from cauda epididymis sperm was performed by computer-assisted sperm analysis (CASA) using CEROS II system equipped with miniTherm stage warmer (Hamilton-Thorne, Bervely, MA, USA). For sperm isolation, cauda epididymis was dissected, rinsed with 0.1 M PBS pH 7.4, and placed in 3 ml of HTF medium (Irvine Scientific, Santa Ana, CA, USA) supplemented with 0.75% BSA at 37 °C under 5% CO_2_ and air. Four to six holes were carefully made in the cauda epididymis using a 30-G needle, allowing sperm release into the medium. After 10 min of incubation, tissue was removed and aliquots of sperm suspension were diluted 1:20 to 1:30 into fresh medium to a concentration of ~2 × 10^5^ sperm/ml. A 15-μl aliquot of diluted sperm suspension was placed onto 80- μm 2XCELL slides (Hamilton Thorne) for motility assessment. Sperm tracks were captured at 37 °C with a frame rate acquisition of 60 Hz for 1 s (60 frames captured) and 4X negative-phase contrast objective using default instrument settings. We recorded at least 200 spermatozoa and 8 fields for each sample analyzed. We used the video playback to remove mistracked spermatozoa, as may occur due to collisions and false-negative debris detection. We recorded sperm tracks and kinematics parameters individually and removed sperm tracks with less than 30 acquisition points from subsequent analysis^48^. We evaluated the following sperm kinematics parameters as provided by the CASA system: average path velocity (VAP; µm/s), straight line velocity (VSL; µm/s), curvilinear velocity (VCL; µm/s), lateral head displacement (AHL; µm/s), straightness (STR; %), and linearity (LIN; %). Sperm tracks were classified as motile, progressive, slow and static; motile tracks were classified as progressive if STR ≥ 65% and VAP ≥ 100 µm/s, and slow if VAP ≤ 20 µm/s and VSL ≤ 30 µm/s.

For evaluation of sperm morphology, we prepared air-dried smears onto microscope slides using fresh sperm suspension. Slides were fixed and then stained using panoptic dye as previously described^49^. We examined two hundred cells per slide and individually scored them as normal or abnormal (straight heads, isolated heads and flagellums), according to the strict sperm morphology criteria.

### Determination of plasma testosterone concentration

Plasma testosterone concentration was measured by ELISA using IBL testosterone (rat/mouse) ELISA kit (IBL International GMBH, Hamburg, Germany) according to the manufacturer’s instructions. The assay detection limit was 70 pg/ml and the detection range was 0.20–16.0 ng/ml.

### Statistical analysis

Results were expressed as mean ± SEM or median from the indicated number of animals per group. Data from RT-qPCR were compared by Student’s *t*-test (two groups) or by One-way analysis of variance (ANOVA) followed by Dunnet test (more than two groups). Multiplex bead assay and quantitative sperm results were compared by ANOVA followed by Bonferroni test. Percentage data from sperm morphological evaluation were transformed to logarithm values previous to statistical analysis. Hormonal data were compared by Kruskal-Wallis followed by Dunn test. Statistical analyses were performed using GraphPad Prism^®^ 5.0 software (GraphPad Software Inc., San Diego, CA, USA). *p* < 0.05 was considered statistically significant.

### Data availability

All data generated during this study are included in this published article (and its Supplementary Information files).

## Electronic supplementary material

Supplementary Information
