# Supplementary material for: Lipopolysaccharide and lipotheicoic acid differentially modulate epididymal cytokine and chemokine profiles and sperm parameters in experimental acute epididymitis
Source: Sci Rep. 2018 Jan 8;8:103. doi: 10.1038/s41598-017-17944-4 (PMC5758752; doi:10.1038/s41598-017-17944-4)
Supplement: Supplementary file 1 — Supplementary Information [file 41598_2017_17944_MOESM1_ESM.pdf]

Supplementary Materials

Lipopolysaccharide and lipotheicoic acid differentially modulate epididymal cytokine and chemokine profiles and sperm parameters in experimental acute epididymitis

Erick J.R. Silva[1,2,*], Camilla M. Ribeiro[1], André F. M. Mirim[1], Alan A. S. Silva[2], Renata M. Romano[1,6], Jorge Hallak[3,4], Maria Christina W. Avellar[1,*]

Affiliations:

[1]Section of Experimental Endocrinology, Department of Pharmacology, Universidade Federal de São Paulo - Escola Paulista de Medicina, São Paulo, SP, Brazil, 04044-020.

[2] Department of Pharmacology, Institute of Biosciences of Botucatu, Universidade Estadual Paulista "Júlio de Mesquita Filho", Botucatu, SP, Brazil, 18618-869.

[3] Androscience, Science and Innovation Center in Andrology, São Paulo, SP, Brazil, 03178-200.

[4] Reproductive Toxicology Unity, Department of Pathology and Division of Urology, Hospital das Clínicas, University of São Paulo Medical School, São Paulo, SP, Brazil, 01246-903.

[*]Corresponding authors: M. C. W. Avellar, e-mail avellar@unifesp.br, phone number +55 11 5576-4448 or Erick J. R. Silva, e-mail ejrsilva@ibb.unesp.br, phone number +55 14 3880-0223.

[6]Current Address: Department of Pharmacy, State University of Centro-Oeste, Guarapuava, PR, Brazil, 85040-080.

Supplementary Results

Distribution of Blue Evans Dye into the epididymis after intravasal injection

Since the rat epididymis is highly segmented by connective tissue septa[1], which could provide a physical barrier to ascending pathogens[2], we initially performed experiments using saline solution containing Blue Evans tracer injected into the vas deferens to demonstrate its distribution into the cauda epididymis (Supplementary Fig. S1). After 30 min, we observed intense blue staining in the distal region of the cauda epididymis (regions 18-19 of the rat epididymis[1]), which became faint in the proximal cauda region (regions 15-17) and not detectable in any other proximal region of the organ (Supplementary Fig. S1). These results demonstrated that the ascent of the dye was limited to the cauda epididymis, which is the major epididymal region affected by LPS- and LTA-induced inflammation in our experimental conditions.

Effects of LPS- and LTA-induced epididymitis on the morphology of the epididymis

We investigated the presence of histological signs of epididymal inflammation following 6 h and 24 h of 25 μg LPS or 125 μg LTA intravasal treatments. Epididymides from saline-control rats showed normal morphology with intact epithelial and interstitial compartments, and luminal compartment filled with spermatozoa (Supplementary Figs. S2-S3). On the other hand, we observed an ongoing acute inflammatory reaction in samples from LPS- and LTA-treated rats (Supplementary Fig. S2-S3). The inflammatory signs were more evident in the cauda epididymis (Supplementary Fig. S2), less intense in the distal corpus and absent in the proximal regions of the organ (Supplementary Fig. S3). Cauda epididymis from both LPS- and LTA-treated rats showed intense interstitial inflammatory cell infiltrate and edema at 6 h (Supplementary Fig. S2). Some of the inflammatory cells present in the interstitial space of the cauda epididymis from both LPS- and LTA-treated rats were identified as polymorphonuclear leukocytes based on their nuclear morphology (Supplementary Fig. S2, insets). The inflammatory cell infiltrate in the cauda epididymis was reduced 24 h after LPS or LTA intravasal injection (Supplementary Fig. S2).

Supplementary Discussion

On the rationale for LPS and LTA dose selection

Because different LPS serotypes have different ability to induce inflammation, we selected the doses of ultrapure LPS based on its biological activity, measured as endotoxin units (EU) (1 ng ultrapure LPS from *E. coli* 055:B55 = 10 EU, as informed by the manufacturer, Innaxon, Cat# IAX-100-013). Thus, we employed ultrapure LPS doses equivalent to 50,000 (5 µg), 125,000 (12.5 µg) and 250,000 (25 µg) EU per epididymis. The highest dose we used was 4-8 fold lower than the LPS dose, in EU, used to induce epididymitis via endotoxin injection into the interstitial compartment of the rat caput epididymis[3,4], indicating the sensitivity of our experimental model to trigger an acute inflammatory response in the rat epididymis. Since a similar rationale of dose-selection could not be used for LTA, we used LPS doses as reference to investigate its acute effects in the epididymis. Despite the difficulty to directly compare LPS and LTA, due to their different chemical and biological nature, we compared the effects of both PAMP treatments on the mRNA levels of the inflammatory markers *Il1b* and *Tnf* (used as readouts) to establish their optimal dose to induce inflammation in the cauda epididymis. Our results showed that only *Il1b* mRNA levels could be used as a readout, since LTA did not change the expression of *Tnf* transcripts at any dose analyzed. We could not explore highest LTA doses due to limitations on the volume of solution that could be injected into the vas deferens. Nevertheless, our approach allowed us to determine optimal LPS (25 µg) and LTA (125 µg) doses capable to induce an inflammatory reaction in the cauda epididymis at comparable levels based on *Il1b* mRNA changes. The presence of similar macroscopic and histological displays of inflammation in both LPS- and LTA-treated rats at the same time-point further confirmed an ongoing acute epididymitis.

Supplementary References

1 Jelinsky, S. A. *et al.* The rat epididymal transcriptome: comparison of segmental gene expression in the rat and mouse epididymides. *Biol Reprod* **76**, 561-570, doi:10.1095/biolreprod.106.057323 (2007).

2 Stammler, A. *et al.* Epididymitis: ascending infection restricted by segmental boundaries. *Human Reprod* **30**, 1557-1565, doi:10.1093/humrep/dev112 (2015).

3 Palladino, M. A., Mallonga, T. A. & Mishra, M. S. Messenger RNA (mRNA) Expression for the Antimicrobial Peptides β-Defensin-1 and β-Defensin-2 in the Male Rat Reproductive Tract: β-Defensin-1 mRNA in Initial Segment and Caput Epididymidis Is Regulated by

Androgens and Not Bacterial Lipopolysaccharides. *Biol Reprod* **68**, 509-515, doi:10.1095/biolreprod.102.008953 (2003).
4 Cao, D. *et al.* Lipopolysaccharide-induced epididymitis disrupts epididymal beta-defensin expression and inhibits sperm motility in rats. *Biol Reprod* **83**, 1064-1070, doi:10.1095/biolreprod.109.082180 (2010).

Supplementary Table S1

Concentration of cytokines and chemokines in the cauda epididymis from saline-control, LPS- and LTA-treated rats.

Cytokine/Chemokine Concentration (pg/mg of protein)	Experimental Groups					
	6 h			24 h		
	Saline-control (n=8)	LPS 25 µg (n=9)	LTA 125 µg (n=9)	Saline-control (n=8)	LPS 25 µg (n=7)	LTA 125 µg (n=9)
IL1A	35.0 ± 4.6	78.4 ± 10.7*	37.1 ± 4.1	43.8 ± 5.9	26.4 ± 6.7	26.7 ± 4.7
IL1B	107.8 ± 14.2	326.2 ± 28.8*	156.2 ± 26.4	97.0 ± 20.2	115.2 ± 19.3	83.1 ± 9.1
IL6	653.9 ± 67.5	1211.0 ± 109.1*	945.0 ± 160.1	570.5 ± 113.2	486.8 ± 78.9	494.7 ± 58.5
IL17A	13.1 ± 1.6	13.0 ± 0.9	16.7 ± 3.0	15.4 ± 4.1	9.6 ± 2.5	8.9 ± 1.2
CXCL8	292.0 ± 17.6	279.1 ± 19.7	350.9 ± 48.2	257.6 ± 16.0	284.9 ± 56.1	266.0 ± 29.2
CCL5	462.0 ± 46.3	342.6 ± 31.7	576.2 ± 79.3	433.1 ± 62.0	275.6 ± 38.9	314.6 ± 36.3
CCL2	103.3 ± 12.1	1294.0 ± 165.0*	223.4 ± 42.6*	129.5 ± 11.2	133.1 ± 39.3	100.4 ± 15.7
CSF2	21.3 ± 2.5	18.3 ± 2.6	27.3 ± 5.1	20.5 ± 6.0	14.9 ± 3.2	15.1 ± 1.8
CXCL2	34.6 ± 3.6	65.1 ± 10.8*	51.5 ± 6.5*	31.7 ± 5.7	26.7 ± 4.8	26.8 ± 2.5
IL10	29.0 ± 3.8	44.6 ± 5.8*	46.5 ± 6.3*	23.5 ± 4.4	23.7 ± 3.6	23.1 ± 3.0

Data represent mean ± SEM of experiments performed with samples from the indicated number of rats.

Each sample was normalized to its respective total protein concentration and expressed as µg/mg of protein.

Asterisks indicate statistically different from the respective saline-control group (ANOVA followed by Bonferroni test, $p<0.05$)

Supplementary Table S2

Effect of intravasal LPS or LTA injection on kinematics parameters of spermatozoa from the cauda epididymis from 7-day saline-(control), LPS- and LTA-treated rats.

Experimental Groups	Sperm tracks	Sperm kinematics parameters					
		VAP (µm/s)	VSL (µm/s)	VCL (µm/s)	ALH (µm/s)	STR (%)	LIN (%)
7-day saline	2087	134.9 ± 12.9	92.3 ± 17.2	306.1 ± 36.6	23.9 ± 3.0	69.5 ± 10.8	31.0 ± 5.4
7-day LPS	1097	152.6 ± 17.8	103.0 ± 19.3	355.5 ± 38.0	24.8 ± 2.8	68.9 ± 10.4	29.9 ± 5.7
7-day LTA	1747	153.4 ± 21.0	109.9 ± 20.7	359.7 ± 36.8	24.6 ± 2.8	73.0 ± 9.8	31.7 ± 5.5

Data represent mean ± SEM from experiments performed with samples from 6 rats/group. The total number of sperm tracks analyzed is indicated.

VAP= average path velocity; VSL= straight line velocity; VCL = curvilinear velocity; ALH = amplitude of lateral head displacement; STR = straightness; LIN = linearity.

Supplementary Table S3

Oligonucleotide sequences used in qPCR studies. The accession number, each oligonucleotide pair sequences, amplicon size (in base pairs, bp) and correspondent amplification efficiency are indicated for each gene.

Transcript	Accession number	Sequences (5´-3´)	Amplicon size (bp)	Efficiency (%)
Il1b	NM_031512	CCAGGATGAGGACCCAAGCA<br>TCCCGACCATTGCTGTTTCC	510	96
Tnf	NM_012675	TGCCTCAGCCTCTTCTCATT<br>GCTTGGTGGTTTGCTACGA	209	105
Il6	NM_012589	GCGATGATGCACTGTCAGAA<br>AGCATTGGAAGTTGGGGTAGG	281	103
Infg	NM_138880	CAGGCCATCAGCAACAACATA<br>AGCACCGACTCCTTTTCCGCT	214	97
Nos2	NM_012611	CGGAGAACAGCAGAGTTGGT<br>TTGTGGTGAAGGGTGTCGTG	341	100
Ptgs1	NM_017043	CCTCACCAGTCATTCCCTGT<br>TATCCGTGTGTCAGCAGGAA	202	95
Ptgs2	NM_017232	TGGGTGTGAAAGGAAATAAGG<br>AGGATACACCTCTCCACCGA	302	102
Nfkbia	NM_001105720	CATGAAGAGAAGACACTGACCATGGAA<br>TGGATAGAGGCTAAGTGTAGACACG	329	96
Il10	NM_012854	AGACCCTCTGGATACAGCTG<br>GCTCTATTTATGTCCTGCAG	228	96
Il4	NM_201270	CCGAGATGTTTGTACCAGAC<br>AGTGTTGTGAGCGTGGACTC	229	97
Ppia	NM_017101	AGCACTGGGGAGAAAGGATT<br>GATGCCAGGACCTGTATGCT	174	90

Supplementary Figures

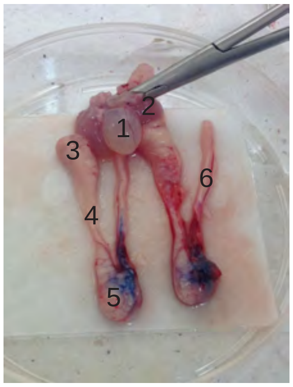

Supplementary Fig. S1 Distribution of Blue Evans dye to the rat cauda epididymis 30 minutes after its retrograde injection into the lumen of the epididymial portion of the vas deferens. 1: bladder, 2: ventral prostate, 3: caput epididymis, 4: corpus epididymis, 5: cauda epididymis, 6: vas deferens.

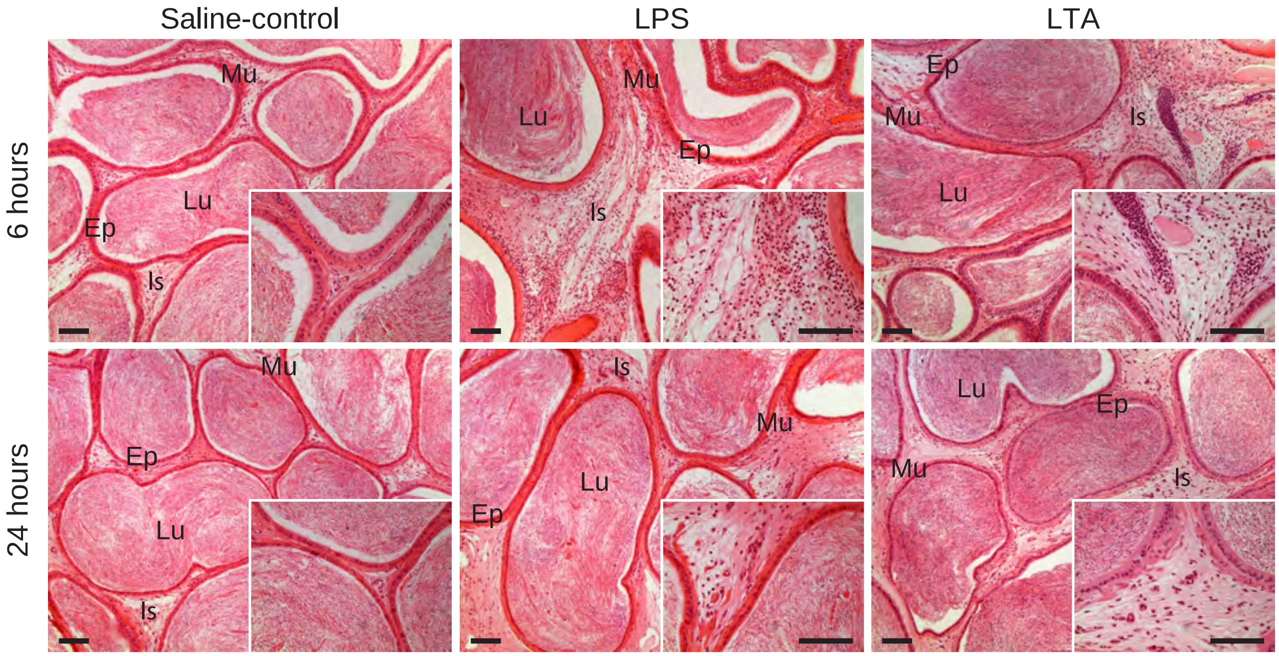

Supplementary Fig. S2 Effects of intravasal LPS (25 μg) or LTA (125 μg) treatment on the morphology of the cauda epididymis. Representative photomicrographs of epididymides collected from saline-control, and LPS-treated rats euthanized 6 h and 24 h after treatment. Epididymal cross sections were stained with hematoxylin/eosin. Results are representative of experiments performed with tissues from three rats per group. Ep: epithelium, Is: interstitial space; Lu: lumen; Mu: smooth muscle. Scale bar = 100 μm.

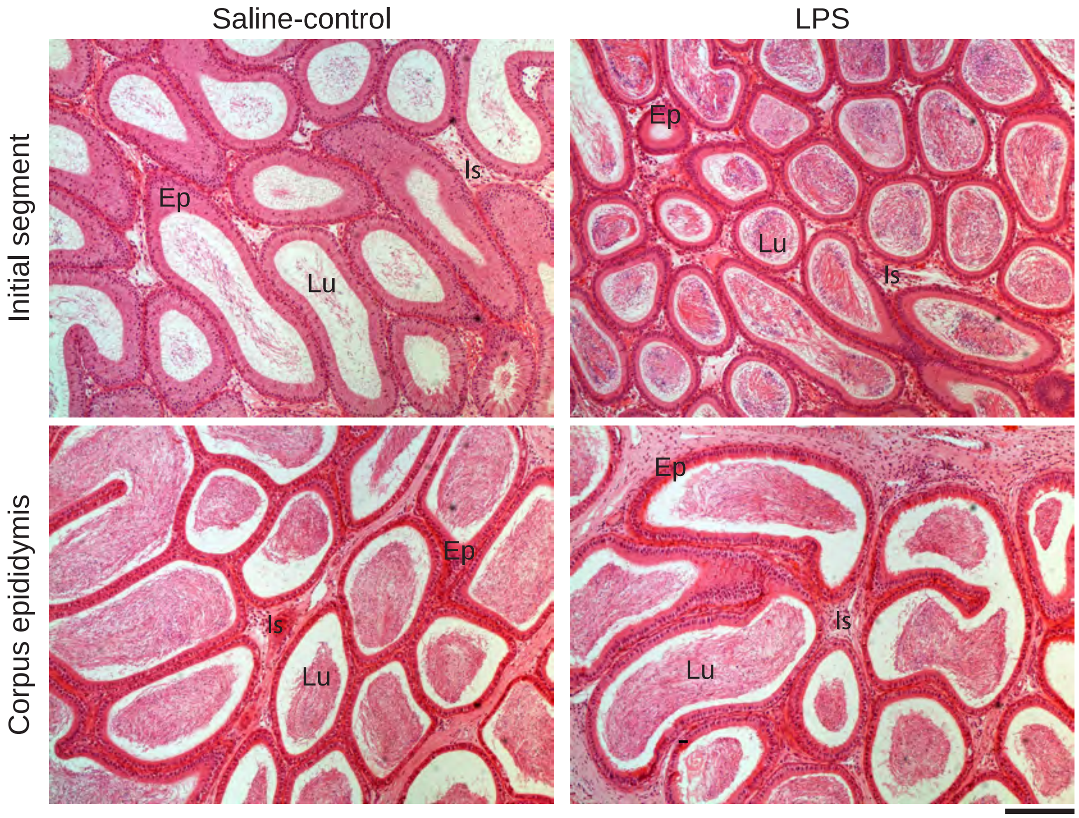

Supplementary Fig. S3 Effects of intravasal LPS (25 µg) treatment on the morphology of the initial segment and corpus epididymis. Representative photomicrographs of epididymides collected from saline-control, and LPS-treated rats euthanized 6 h and 24 h after treatment. Epididymal cross sections were stained with hematoxylin/eosin. Results are representative of experiments performed with tissues from three rats per group. Ep: epithelium, Is: interstitial space; Lu: lumen. Scale bar = 200 µm.

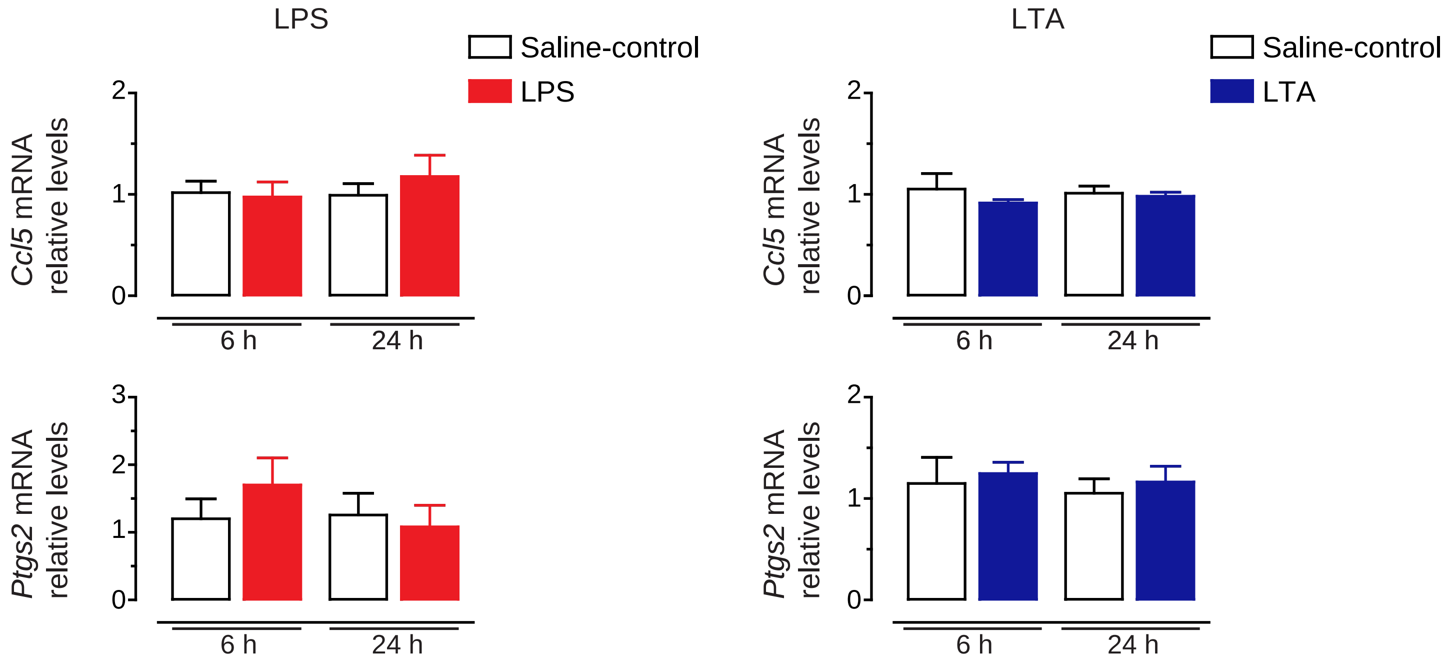

Supplementary Fig. S4 Effect of intravasal LPS (25 µg) or LTA (125 µg) treatment on the mRNA levels of *Ccl5* and *Ptgs1* transcripts by RT-qPCR in the cauda epididymis from saline-control, LPS- or LTA-treated rats euthanized 6 h or 24 h after treatment. Samples were normalized to their internal control (Ppia) and expressed as relative values of their respective saline-control groups. Results are expressed as mean ± SEM of experiments performed in duplicate with samples described in Fig. 2.

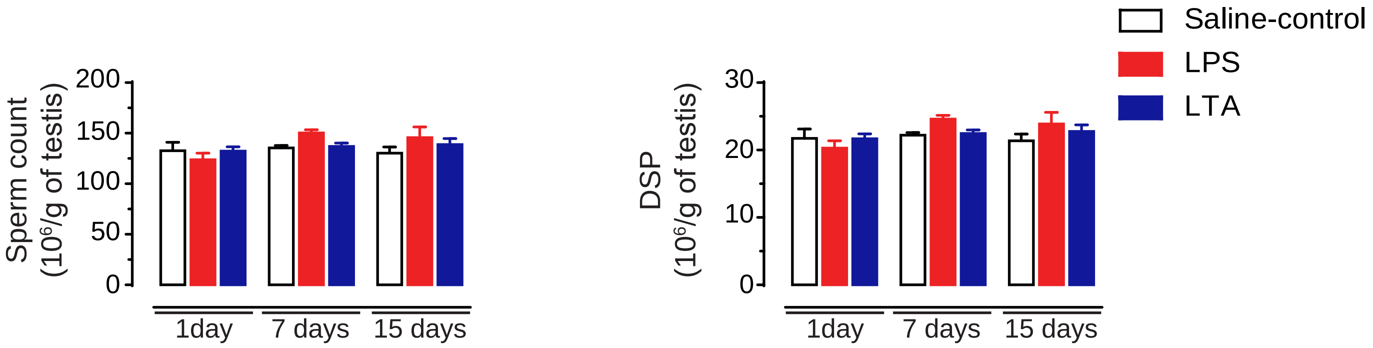

Supplementary Fig. S5 Effects of intravasal LPS (25 µg) or LTA (125 µg) on testicular sperm count daily sperm production (DSP). Testes were collected from saline-control, LPS- or LTA-treated groups euthanized 1, 7 or 15 days post-treatment. Results are expressed as mean ± SEM of experiments performed with samples from six (1-day LPS and 1-day LTA), seven (1-day saline-control), and eight (7-day and 15-day saline-control, 7-day and 15-day LPS, and 7-day and 15-day LTA) rats per group.

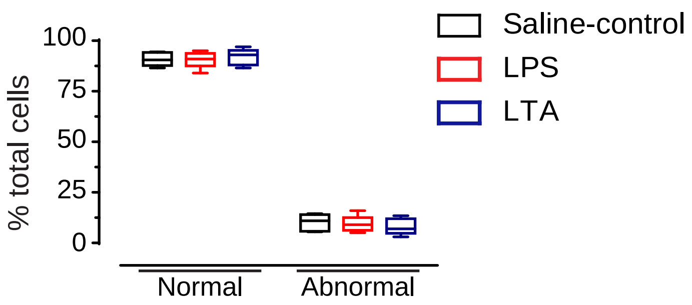

Supplementary Fig. S6 Effects of intravasal LPS (25 μg) or LTA (125 μg) on sperm morphology. Spermatozoa were isolated from the cauda epididymis from saline-control, LPS- or LTA-treated groups euthanized 7 days post-treatment. Box plots (median, interquartile range and whiskers min-max) represent the percentage of motile, progressive and static spermatozoa. Experiments were performed with samples from six rats per group.
